# Understanding public trust in national electronic health record systems: A multi-national qualitative research study

**DOI:** 10.1177/20552076251333576

**Published:** 2025-04-03

**Authors:** Kimon Papadopoulos, Elske Ammenwerth, Guillaume Lame, Nina Stahl, Verena Struckmann, Viktor von Wyl, Felix Gille

**Affiliations:** 1 Digital Society Initiative (DSI), 30841 University of Zürich, Zurich, Switzerland; 2 Institute for Implementation Science in Health Care (IfIS), 30841 University of Zürich, Zurich, Switzerland; 3 Institute of Medical Informatics, 31510 UMIT TIROL Private University for Health Sciences and Technology GmbH, Hall, Austria; 4 Laboratoire de Genie Industriel, 435940 Universite Paris-Saclay CentraleSupelec, Gif-sur-Yvette, France; 5 Department of Health, Ethics and Society (HES), 5211 University of Maastricht, Masstricht, The Netherlands; 6 Department of Health Care Management, 26524 Technical University of Berlin, Berlin, Germany

**Keywords:** Public trust, trust, electronic health record, patient data, qualitative research, Europe, health systems, digital health, eHealth, implementation

## Abstract

**Objective:**

Having public trust in national electronic health record systems (NEHRs) is crucial for the successful implementation and participation of NEHRs within a nations healthcare system. Yet, a lack of conceptual clarity precludes healthcare policymakers from incorporating trust to the fullest extent possible. In response, this study seeks to validate an existing framework for public trust in the healthcare system, which will help provide a clearer understanding of what constitutes public trust in NEHRs across members of the public in different countries, cultures, and contexts.

**Methods:**

Twenty-four focus groups were conducted in Austria, Germany, France, Italy, the Netherlands, and Switzerland with residents of each respective country to discuss their viewpoints on our public trust in NEHRs framework in order to validate said framework.

**Results:**

Frameworks describing the causes and effects of public trust in NEHRs were created for each country studied. Across all countries, the frameworks remained similar to our base framework, highlighting our frameworks’ robustness. Data security, privacy, and autonomy were consistently described as the most important aspects of public trust in NEHRs. Concurrently, health system actors, such as doctors, were found to have significant influence on NEHR implementation. Their influence, however, can either be beneficial or detrimental to public trust in NEHRs, depending on their actions and how the public perceives those actions. Additional results detail contextual insights into country-specific viewpoints and the role of healthcare stakeholders in public trust in NEHRs. The results showcase the differences and similarities in which different populations across Europe view trust in NEHRs in the context of our framework.

**Conclusions:**

These findings present public trust frameworks in the context of NEHRs for the study countries. These frameworks can assist stakeholders in obtaining a comprehensive understanding of the complexity of public trust in implementing and promoting their NEHRs, including measurements of public trust.

## Introduction

National electronic health record systems (NEHRs) are dedicated digital repositories of an individual's healthcare information and history that are consolidated, implemented, and supported by a national government.^1^ Since the mid-1990s, these NEHRs are becoming of increasing importance to both public health and healthcare in the European region and across the globe.^2–4^ This is due to electronic health records (EHRs) bringing a variety of clinical, organizational, and societal benefits when implemented. These benefits provide advancements in both the quality and efficiency of the healthcare system by reducing medical costs and errors, improving patient healthcare engagement, aiding in emergency situations and public health investigations, and facilitating comprehensible and informed communication between patient and physician, just to name a few.^3,5–11^ On account of these benefits, many countries have successfully implemented NEHRs, such as Austria, Estonia, and Sweden.^2,12,13^ These systems can only be successfully implemented and sustained, however, if the public trusts NEHRs. If the public does not trust their NEHRs, healthcare stakeholders lack the public legitimacy to implement NEHRs, and the public will not participate in their NEHR program.^14–18^ As more and more countries around Europe seek to introduce EHRs into their national healthcare systems to bring the advantages of NEHRs to their societies, healthcare stakeholders, including national governments, must understand the trust relationship between the public and NEHR providers to ensure that the public trusts their NEHRs.^3,14,19,20^

Trust in itself can be summarized as a “bet” on the future actions of others, while taking past experiences, current perceptions, and prospective anticipations into account.^20,21^ Public trust can be aptly described as the general public's expectation for institutions, organizations, and individuals that manage, oversee, and/or provide services to them, to act benevolently in the best interests of the public.^22^ These actors must prove these intentions through their actions, which must show integrity, reliability, transparency, and competence. Without this, adequate levels of public trust will be unattainable, and stakeholders cannot operate efficiently to implement long-standing, effective change and they will ultimately face issues with public participation.^3,22^ Therefore, public trust constitutes an indispensable component for the successful implementation of healthcare and public health interventions within a healthcare system, including NEHRs.^23,24^

Research investigating public trust in NEHRs is limited, leading to a lack of conceptual clarity on the topic. Within Europe, variations between the healthcare systems across the continent adds further complexity to determining what is public trust in NEHRs.^14,24^ Providing clarification on the meaning of public trust in the context of NEHRs is essential to enabling a better understanding of how to build and/or maintain trust on this issue; which in turn is instrumental in formulating effective policies and health system actions that ensure the successful implementation of NEHRs into national healthcare frameworks.^18^ Additionally, conceptual clarity can also be utilized to help build public acceptance in NEHR and health data sharing more broadly, inform system design, health policymaking, and international health data transfer, while also serving as a basis for measurements of public trust levels.^3,18,19,25–27^ Having a comprehensive understanding of the concept of public trust in NEHRs is also important in conducting comparative studies on public trust in NEHRs across the European region, which can help inform European Union (EU) policies seeking to implement European-wide EHR systems and cross-border health data transfer.^19,28^ Ultimately, without a common understanding of the concept of public trust in NEHRs, it will be challenging to develop targeted health policies that foster public trust and implement effective comparative measures in assessing public trust levels.^18^

In order to ascertain a clearer understanding of public trust in NEHRs, the objective of this study is to validate an adapted framework for public trust in NEHRs. The framework is based upon a conceptual framework of public trust in the health system in England.^18^ The public trust in NEHRs framework was tested across multiple European countries in order to assess its validity against a plethora of cultures, languages, societies, and contexts.^18^ This is accomplished by transferring the framework to Austria, Germany, France, Italy, the Netherlands, and Switzerland using qualitative research data obtained through focus group interviews in each country. With this data, we developed specific frameworks for each country. Nations can use these tailored frameworks to inform and enhance public trust in their respective national healthcare systems.

### Public trust in the healthcare system: preparatory work

This study utilizes the public trust in the health system framework, which was created previously in the context of the National Health Service (NHS) in England.^20^ This framework is composed of sixteen causal themes describing what builds public trust and two effect themes describing the results of public trust, with the aim to provide guidance in public trust policymaking and public trust measurement.^18,20^ This framework was developed utilizing psychometric principles, a review of previous frameworks of public trust in the healthcare system and trust theory, and an analysis of three qualitative NHS England case studies (care.data, biobank research, and the 100,000 Genomes Project).^18^

The “public trust in the healthcare system” framework was used as a foundation for this study, where it was refined to pertain to public trust in EHRs specifically. Through an inductive and iterative process by KP and FG, the team was able to determine which themes were transferable to the topic of EHRs, and then reiterated each theme in more context-specific language.^29^ In this process, it was determined that the themes of public financial benefit, health system benefit, and gut feeling would be removed. Public financial benefit and health system benefit were found to be similar enough conceptually to the theme of “benefit to others” so these themes were combined into one (benefit to others). Gut feeling was removed as, while it was based in theory that was founded upon the results of the public trust in the healthcare system framework, it was more an extrapolation of the data, not a direct result of the framework development. This resulted in a final framework with thirteen causal themes (themes that allow one to trust EHRs more) and two effect themes (themes that result from public trust in EHRs being present) (Table 1).

**Table 1.** Public trust in NEHRs: causes and effects—framework (adapted from the “public trust in the healthcare system” framework by Gille et al. 2021)

Causal theme	Contextual definition
Active Regulatory Systems	If health system actors who manage and provide EHR systems actively comply with laws and regulations, you then trust EHRs more.
Anonymity	If EHRs anonymize your data, you then trust EHRs more.
Autonomy	If you can decide whether you want to participate in EHRs or not, you then trust EHRs more.
Benefit to Others	If there is a benefit for others and the system through the use of EHRs, you then trust EHRs more.
Certainty about the Future	If those who use EHRs do their best to foresee possible risks in the use of EHRs, then they trust EHRs more.
Familiarity	If you had a positive experience with EHRs, you then trust EHRs.
General Perception of Security	If you consider EHRs safe to use, you then trust EHRs.
Information Quality	When honest and truthful information is communicated about EHRs, there is more trust in EHRs.
Personal Benefit	If you have a personal benefit from EHRs, you then trust EHRs.
Privacy	When EHRs protect your privacy, you trust EHRs.
Recognized Potential of the Healthcare System	If you see a potential in EHRs to achieve what they are introduced for, you then trust EHRs.
Respect	When you and your healthcare provider respect each other, you trust EHRs more.
Time	If you are given sufficient time to decide whether you want to participate in EHRs or not, you trust EHRs more.

**Effect theme**	**Contextual definition**
Participation	If you trust EHRs, then you use EHRs.
Legitimacy	If you trust EHRs, health system actors have the authority to implement EHRs.

## Methods

**Figure 1.**
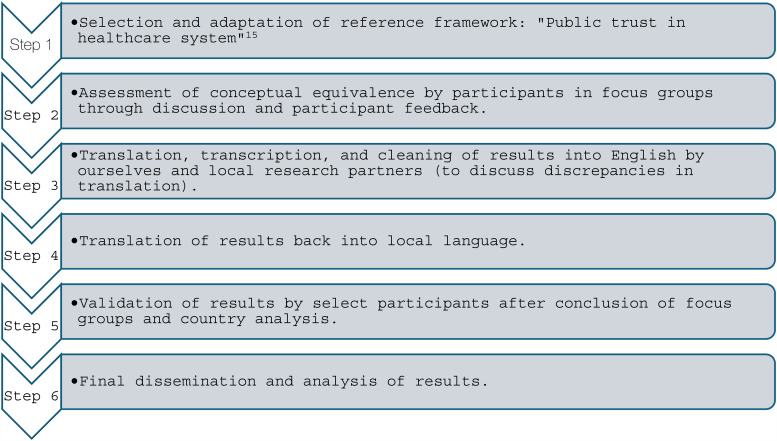
Process and analysis of focus group interviews.

Between October 2022 and June 2023 (full timeline in Table 2), KP and FG conducted 24 focus groups with overall 89 members of the public in Austria, France, Germany, Italy, the Netherlands, and Switzerland, with four focus groups per country. These focus groups were conducted to validate the public trust in NEHRs framework for each country. A step-by-step process, following Sidani et al.’, 2010, cultural adaptation methodology, of all methodological steps conducted in this study can be seen in Figure 1.^30^ This study adhered to the COREQ checklist for qualitative interviews and focus groups.^31^

**Table 2.** Data collection information.

Country	Partner university	Collaborators	Month(s) and year of data collection
Austria	UMIT Tirol—Private Universität für Gesundheitswissenschaften und -technologie	Prof. Elske Ammenwerth & Stefan Richter	October 2022
Germany	Technische Universität Berlin	Verena Struckmann	February-March 2023
France	CentraleSupélec—Université Paris-Saclay	Prof. Guillaume Lame	April 2023
Italy	Università di Torino	Prof. Antonio Santangelo & Prof. Simona Stano	March 2023
The Netherlands	Universiteit Maastricht	Prof. David Townend, Birgit Wouters, & Nina Stahl	June 2023
Switzerland	Universität Zürich	N/A	December 2022

The countries included in the study were selected based upon Switzerland and its neighboring countries, due to cross-border exchange across those countries, as well as the opportunity to explore the variety of languages, cultures, health and government systems, and societies of each country. These countries were also selected due to their proximity to the core research team, based in Switzerland, facilitating easier access to study populations as all countries include or border the research teams’ base of operations. At the start of data collection, the research team decided to expand the scope of the project and sought to include a higher variety of countries to the study. Overtures were made in Sweden, Latvia, the Czech Republic, and the Netherlands, however, due to practical feasibility, only the Netherlands was added to the research study. In Austria, France, Germany, Italy, the Netherlands, and Switzerland, KP and FG established connections and worked with expert academics in universities within each country, who aided in recruitment, establishing country context, and providing support in establishing the ethical requirements necessary to conduct research in each country, when applicable. The list of each partner university can be seen in Table 2.

All focus groups in Austria, Germany, France, Italy, and Switzerland were conducted online using Zoom, while MS Teams was used in the Netherlands. Originally, focus groups were to be performed with two online sessions and two in-person sessions. However, it was decided to conduct all focus groups solely online due to an anticipated lower risk of drop-out from ongoing concerns of COVID-19 (as the study was developed in 2021, during the SARS-CoV-2 pandemic). In addition, the decision to perform strictly online interviews was based on our data collection experience in Austria, the first country where data was collected, where there was considerably lower participant enrollment for the in-person focus groups compared to our online focus groups. As well, online focus groups allowed for a wider geographic sampling of the population in each country, creating the potential for more varied viewpoints from different areas of each country. The same protocol for conducting and reporting the focus groups was used consistently in each of the six countries.

The focus groups were conducted with up to 12 participants per group. The size of the focus groups typically ranged between three and five participants per group. While small, these group sizes allowed each participant to participate in discussions more actively with moderators and with each other, and also provided ample space for one to express their opinion in depth. As well, even with small sample sizes, saturation was continually reached by the third to fourth focus group, an important indicator of a successful focus group, according to previous research, which nullified the need for continued data collection beyond the fourth focus group.^32–34^ Total number of participants per country can be seen in Table 3.

**Table 3.** Demographics—gender and age.

Country	Gender		Age	
Austria *N* = 17	Female	12	18–29	6
	Male	5	30–49	8
	Other	0	50–64	2
	No response	0	65+	0
			No response	1
Germany *N* = 12	Female	7	18–29	5
	Male	5	30–49	3
	Other	0	50–64	0
	No response	0	65+	1
			No response	3
France *N* = 12	Female	6	18–29	8
	Male	6	30–49	4
	Other	0	50–64	0
	No response	0	65+	0
			No response	0
Italy *N* = 26	Female	10	18–29	15
	Male	15	30–49	9
	Other	0	50–64	0
	No response	1	65+	0
			No response	2
The Netherlands *N* = 9	Female	6	18–29	6
	Male	2	30–49	2
	Other	0	50–64	0
	No response	1	65+	0
			No response	1
Switzerland *N* = 13	Female	7	18–29	5
	Male	6	30–49	2
	Other	0	50–64	2
	No response	0	65+	1
			No response	3

With the research team comprising of a native German and English speaker, the focus groups in German-speaking countries (Austria, Germany, and Switzerland) were conducted and moderated in German by FG, with KP co-moderating. In France, Italy, and the Netherlands, focus groups were conducted and moderated in English by KP, with FG co-moderating. For participation in the focus groups, we recruited members of the public who were 18 years of age or older, were a citizen or resident of the country in which the focus group was taking place, and could speak German (in Austria, Germany, and Switzerland) or English (in France, Italy, and the Netherlands). Recruitment for the focus groups was accomplished using different configurations in each country of newspaper advertisement, online flyers sent via social media networks, word of mouth, and support from the local partner universities. Online focus group discussions were conducted over the course of 60 minutes being allocated to the introduction of the topic and the primary group discussion. At the conclusion of the focus group discussion, participants were compensated with €/CHF 30 vouchers to popular, local shopping or grocery chains.

Interviews were audio-recorded using the software QuickTime Player. Focus groups that were conducted in German, were transcribed verbatim in German and then translated into English by FG and KP. English interviews were transcribed verbatim and kept in English. All transcriptions were completed with the transcription function in MS Word.

In these focus groups, we explored and scrutinized what participants thought and felt about public trust in EHRs in relation to the themes presented in the framework. At the beginning of each focus group, participants were introduced to the research team, the concept of public trust, EHRs, and each of the themes in the framework, including definitions. Afterwards, group discussion took place utilizing the online whiteboard software, Flinga, where participants were able to provide initial personal thoughts and feelings regarding public trust in EHRs, before expressing their viewpoints on each theme in regard to its relation and importance to public trust in EHRs (example in Appendix A).^35^ The results from each whiteboard group discussion were then amalgamated through an iterative and inductive process by KP and FG, resulting in the development of an updated framework that provided an overview of each country that was based on participant's feedback and responses to each theme.^29^ Modifications to aspects of the terminology were made within the frameworks to bolster the clarity of certain terms to the public and make certain terms and phrases more easily readable and comprehensible to a wider audience. As well, in five of the six countries studied, certain themes were removed from the framework as participants found these themes did not contribute to increasing public trust in NEHRs. These changes were made based on participant feedback as to the wording and necessity of certain terms, phrases, and themes. Each country's framework was then translated into the local language for each country, utilizing the software DeepL and then cross-checking the translation with native speakers.^36^ The term “EHR” was replaced with the equivalent term in the local language (Austria, ELGA; Germany, ePA; France, DMP; Italy, FSE; Netherlands, EPD; Switzerland, EPD).

Following the group discussions, a brief, optional survey was conducted to gather general, anonymized demographic data: age, gender, country of origin, country/region of residence, educational background, and employment status. Demographic data for age and gender are summarized in Table 3, while full participant demographic data is presented in Appendix B. Afterwards, once all frameworks were completed per country, results were sent to the local colleagues we partnered with, who analyzed and corroborated our results, as well as provided feedback on our translations of the final frameworks into the local language. The results were then sent back to participants in a post-interview survey analysis. In this survey, participants were provided with their country's framework and were asked to provide feedback and/or corrections to the framework as they saw fit. Other than corrections in the syntax and/or grammar of the native translations per country, the result of the participant feedback was positive and no further changes to the frameworks was required or implemented.

## Results

From the focus groups, updated frameworks were developed for each country. Each framework is unique and represents the attitudes, opinions, and feedback of the residents from each country. In these results (Table 4), we highlight the changes each framework went through and describe the difference between the new, country-specific frameworks (Appendix C), and the original framework we began with. Additionally, all novel themes that were added to the frameworks by participants are further described in Table 5.

**Table 4.** Presence of base framework themes in new frameworks developed through focus groups—country-by-country comparative analysis.

Causal themes	Austria	Germany	France	Italy	The Netherlands	Switzerland
Active Regulatory Systems	✓	✓	✓	✓	✓	✓
Anonymity	✓	✓	✓	✓	✓	✓
Autonomy	✓	✓	✓	✓	✓	✓
Benefit to Others	✓	✓	✗	✗	✓	✓
Certainty about the Future	✓	✗	✗	✓	✓	✗
Familiarity	✓	✗	✓	✓	✓	✗
General Perception of Security	✓	✓	✓	✓	✓	✓
Information Quality	✓	✗	✓	✓	✓	✓
Personal Benefit	✓	✓	✗	✗	✗	✓
Privacy	✓	✓	✓	✓	✓	✓
Recognized Potential of the Healthcare System	✓	✓	✗	✗	✓	✓
Respect	✓	✓	✓	✓	✓	✓
Time	✓	✓	✓	✓	✓	✓

**Effect themes**						
Participation	✓	✓	✓	✓	✓	✓
Legitimacy	✓	✗	✓	✓	✓	✗

**Table 5.** New themes added to framework through focus groups.

New causal themes	Contextual definition	Country where theme was added
A system benefit arises from ELGA	If the use of ELGA benefits the healthcare system, then you trust ELGA.	Austria
Reliability of ELGA	If ELGA is a reliable system, then you trust ELGA.	Austria
Time savings through ELGA	If ELGA leads to time savings in the healthcare system, then you trust ELGA.	Austria
Careful data handling within the ePA	If your data is handled carefully, then you trust the ePA.	Germany
Transferability of the ePA between suppliers	If the ePA is transferable between suppliers, then you trust the ePA.	Germany
Easy-to-understand ePA	If the ePA is easy to understand, then you trust the ePA.	Germany
Secure introduction of the ePA	If the ePA introduction is ensured, you trust ePAs.	Germany
User-friendliness of the ePA	If the ePA is user-friendly, then you trust the ePA.	Germany
Data quality in the ePA	If the data in the ePA is correct, then you trust the ePA.	Germany
Recommendation of others to use the EPD	If others recommend the EPD to you, then you trust the EPD.	Switzerland
Uniform communication about the EPD	If communication via the EPD is consistent, then you trust the EPD.	Switzerland

New effect themes		
The ePA is accepted	If you trust the ePA, you then accept the ePA.	Germany / Switzerland
ePA branding through use	If you trust ePAs, its widespread use leads to brand building.	Germany
Research benefits of the ePA	If you trust ePAs, then ePAs can be used for research.	Germany
Positive Feeling of Participation in the EPD	If you trust the EPD, you have a positive feeling when participating in the EPD.	Switzerland
Recommendation of the EPD to others	If I trust the EPD, then I recommend the EPD to others.	Switzerland

### Austria

For Austria, three new causal themes were added: a system benefit arises from ELGA, reliability of ELGA, and time savings through ELGA. No causal themes were removed from the original framework. For the effect themes, the wording for legitimacy and participation were changed, though the core principle behind both concepts remains the same. Otherwise, all changes to themes were specific changes to the language of the terms used for the causal themes, mostly to expand or add clarity to what is being conveyed by the themes.

### Germany

In Germany, six new causal themes were added: careful data handling within the ePA, transferability of the ePA between suppliers, easy-to-understand ePA, secure introduction of the ePA, user-friendliness of the ePA, and data quality in the ePA. As well, three causal themes were removed: certainty of the future, familiarity, and information quality. For effect themes, legitimacy was removed, wording for participation was changed, and three themes were added: the ePA is accepted, ePA branding through use, and research benefits of the ePA. All other changes were specific changes to the language and terms used for the themes following participant suggestions. These were made for the sake of clarity and elaboration.

### France

In France, four causal themes were removed: benefit to others, certainty about the future, personal benefit, and recognized potential of the healthcare system. These themes were removed as they were seen as unimportant to whether one trusts in DMP. No new causal or effect themes were added to the framework. All further changes to themes were specific changes to the language of the terms used for the causal and effect themes, at the behest of participant suggestions, without changing the core meaning of any of the themes.

### Italy

In Italy, three causal themes were removed: benefit to others, personal benefit, and recognized potential of the healthcare system. No new causal or effect themes were added. All remaining changes to themes were to the language of the terms used for both the causal and effect themes. This was completed to add clarity and expansion to certain themes that required it, according to participant feedback.

### The Netherlands

In the Netherlands, one causal theme was removed, personal benefit, and no new causal or effect themes were added. All additional changes to themes were made to provide clearer communication on what is being conveyed by the themes, based upon participant feedback.

### Switzerland

In Switzerland, two causal themes were removed: certainty of the future and familiarity. Additionally, two new causal themes were added: recommendation of others to use the EPD and uniform communication about the EPD. For effect themes, legitimacy was removed, the wording for participation was changed, and three themes were added: the EPD is accepted, positive feeling of participation in the EPD, and recommendation of the EPD to others. All other changes to the framework were specific changes to the language used to convey each theme, utilizing participant feedback on how to better impart the meaning of each theme.

### Additional results

#### Participant viewpoints

While conducting the focus group discussions, each country brought forth a unique viewpoint, opinion, or experience in at least one of the focus groups. These points are noteworthy enough to elicit mention, as they provide context to the study populations, and this contextual data helps to inform the frameworks produced by each study country.

In Austria, participants conveyed high levels of trust in their government and in EHRs, especially when compared to the other countries within the study. As well, unlike most countries, the majority of participants had experience with using an EHR, with most giving positive feedback on how their national EHR system (ELGA) operated.

In Germany, participants expressed concern with EHR implementation for certain populations in Germany, with a particular focus on older residents who lived or were raised in former East Germany. Some participants expressed the opinion that those in former East Germany were more wary of government involvement in their lives, especially when the government will have potential access to private information, such as health information available on an EHR, due to the overall stringent government oversight of the population within the German Democratic Republic (1949–1990).

In France, in comparison to the other study countries, participants had the greatest number of critiques on the topic of public trust in EHRs, in the context of our framework. Over the course of our focus groups in France, participants were particular in how important the framework's themes were to building public trust in EHRs. They expressed that roughly 30% of the presented themes were not important to EHR trust building and did not believe that there were further concepts that could aid in increasing public trust in EHRs. Therefore, those themes were removed, resulting in France having the shortest framework in the study.

In Italy, some participants expressed an especially unique opinion that they had little trust in their own federal government in terms of general trust and in the implementation of a national EHR system; yet, they were more trusting of the EU council and would be more willing to trust and use an EHR that was implemented by the EU instead of the Italian federal government. They expressed that the efficiency and greater oversight in the EU made them feel that their data would be more secure in an EU-provided EHR, and that implementation would be conducted in a more optimal approach than the Italian government would be capable of.

In the Netherlands, participants across multiple focus groups expressed general distrust and concern with their government having access to the personal, private health information available on an EHR due to various scandals involving the Dutch government and data mismanagement. They stated that previous scandals have eroded trust in the system, and that the government would need to take significant steps in order to prove to its citizens that such data breaches would not occur with the frequency they had in the past.

In Switzerland, the current approach to EHR implementation was reported by interviewees to be quite fractured and confusing to the average person. This level of perplexion exasperates the public enough that they are unsure or are unmotivated to participate in using EHR. This even extends to physicians in Switzerland. In one focus group, a participant shared with moderators that they sought to enroll in an EHR program in her canton, however, there were eight options from which to choose from, leading her to be unsure of which one to enroll in. When they went to their physician to ask for assistance and clarity in choosing the best EHR provider, their physician recommended that they not even bother with enrolling in an EHR at the present moment, as it was not worth the hassle in their opinion. The participant stated that if their own doctor did not advocate for enrolling in an EHR, they were not only disinclined from using one, but also lost trust in both EHRs and the current approach to EHRs in Switzerland.

#### Influence of health system actors

An interesting finding from the data produced by this cross-country comparison is the importance both healthcare and political actors have in influencing the public's trust. As can be seen in the previous section, actors, such as doctors and governments, sway the level to which the public will trust something. In all study countries, except for France, participants gave personal or hypothetical examples of how past actions of and/or the current attitudes towards health system actors have either fostered, or, in our case, more commonly, diminished public trust in their NEHRs.

## Discussion

Through validation of the public trust in NEHRs framework, we gain a clearer understanding on what constitutes public trust in EHRs for the general public in a variety of countries, cultures, and contexts across parts of western, central, and southern Europe. As the original public trust in the healthcare system framework that served as a basis for this study was relative only to England, putting the framework into practice outside of England and seeing how it held up across public participants in multiple different countries provided us with many insights; and gave us the opportunity to define different nuances of trust across these countries.

Across all focus groups, there were changes that were consistent and required us to reevaluate certain aspects of the framework. One change was to elaborate on how anonymity plays a role in public trust in EHRs. While we discussed what anonymity within an EHR was in the context of research purposes, we consistently saw that that was unclear to participants across all countries, necessitating a change to how to convey that theme. As a result, anonymity was expanded to “anonymity in research,” to bring clarity to the theme. Another consistent change was to the theme of respect. In our context, “respect” was directed at the respect given between a patient and their doctor, which was not sufficiently clear to participants. Respect was therefore expanded upon to “patient–doctor relationship,” in order to provide clarification on the concept. A third consistent change was made to the theme of time. While not all countries ended up with the same changes, all found that the concept of “time” towards trust, in the context of the original framework, as vague and inconsequential. Consequently, the theme was changed, with the most popular edit being a specification to the time allotted in the decision-making processes, as that aspect of time was seen as a critical component to having trust in a national EHR system. Lastly, the effect theme of legitimacy was another theme where change was necessary based on feedback across all focus groups. Describing the effect that trust has on contributing towards the legitimacy of stakeholders in enacting positive change in the healthcare system, with our focus being on the implementation of national EHRs, was very confusing to participants and required nuanced elaboration on part of the moderators. Therefore, “legitimacy” was reworked across all frameworks, though the nature of these changes varied from country to country, where in some cases it was reworked to the specifications of the participants and in others it was removed entirely.

As can be seen from these persistent issues with the framework, the primary challenge brought forth was in the of clarity of the terms. Although the terms were originally based on previous research and designed to be simple and broad, the original terms were not developed to communicate these themes directly to the public. This approach led to misunderstandings and inconsistencies in interpretation among the members of the public who participated in our focus groups. The feedback indicates a need for more precise and well-defined terminology to avoid confusion when used with everyday people. However, though the terminology was the central issue of the framework, the fact that the core concepts behind the vast majority of the themes were unmodified means we can affirm that the original framework is relatively robust and provides a good basis for conceptualizing public trust in EHRs.

In conjunction with the changes to the framework, there were other themes that were consistent amongst the focus groups and were persistently presented as significant factors to trust building in the context of public trust in NEHRs. The themes of data security, privacy, and autonomy were consistently rated as the most significant drivers of trust in NEHRs by participants across all countries and focus groups. This coincides with and is supported by our previous research into public trust in NEHRs across the available literature ranging from 1995 to 2021.^3^ Participants conveyed the importance of these concepts towards the public having trust in a national EHR system, and that if even one of these aspects were lacking, it would negatively influence public trust in EHRs; an issue of great consequence, as trust, while hard to maintain and build, is easy to lose.

As well, there existed a noticeable trend of apathy or ignorance towards EHRs in most countries, with Austria, the only country in the study with a long-term national EHR system currently in place, being a notable exception. The degree of apathy and ignorance varied, however, from country to country and from person to person. This suggests varying levels of awareness and engagement with national EHR systems, potentially influenced by the various personal experiences, national policies, national politics, healthcare infrastructure, public awareness campaigns, and cultural contexts that come forth from an international study covering multiple countries and populations.

### Actors

Previous research shows that political and medical actors are pivotal in building public trust.^3,37,38^ Their influence on the trust relationship between the public and EHRs extends across multiple facets, with the most significant being the privileged doctor–patient relationship and the social contract that exists between the public and public institutions. These high-impact actors also play a role in ensuring accountability in the governance of health data, which is especially pertinent within a national EHR system.

For governmental/political actors, such as policymakers, providing effective administration and legislation, productive and efficient implementation of a national EHR program, and capitalizing on existing public trust can position the government as a key player in fostering public trust in EHRs.^39–41^ In practical terms, government and political actors can accomplish this with transparent policy design, clear communication on the benefits of EHRs, engagement with the public and incorporating public feedback into system design, and demonstrating accountability via oversight, proper governance, and legal and regulatory frameworks.^3,24,42^ Having an effective legal and regulatory environment plays a crucial role in fostering public trust by both reinforcing the perception of a robust regulatory system that upholds the rule of law, and by enabling policymakers to demonstrate trust-building measures during implementation.^24^ This is especially pertinent for populations that have experienced deficiencies in government responsibility, such as data breaches due to inadequate security measures and protocols, and subsequently have lower levels of trust.^43^ Additionally, policymakers can engage physicians as advocates for EHR adoption by ensuring digital health literacy amongst doctors and offering incentives for providing high-quality, structured data at the source. In conjunction, demonstrating tangible use cases with clear benefits for both doctors and their patients will promote active participation and support, and will encourage doctors to be advocates for NEHR adoption and use.^19^

A doctor's influence has the deepest impact on public trust in a national EHR system, though this influence is context-specific. Clinicians are among the most trusted professions. They have a direct relationship with patients, and they also often help patients navigate through the health system. As such, they easily gain the trust of the public and have an influential position over their patients’ healthcare decisions, acting as opinion multipliers. Their direct experiences with EHRs and interactions with patients give them a good overview of the practical challenges and real-world potential of EHRs and can influence the public's trust of EHRs and NEHRs.^39,40,44–47^ Doctors influence public trust in NEHRs by shaping public perceptions of EHRs through their own views on the national system, either encouraging or discouraging patient participation. Using a data point collected in Switzerland during our focus groups as an example, a doctor who perceives that the costs outweigh the benefits in an EHR system that is not properly implemented, they will persuade their patient not to enroll in the EHR system. However, the opposite can be true if a person's doctor looks favorably upon the national EHR system put in place, which can only happen if the national EHR system is constructed and implemented with the public's best interest at heart.^48^ The factors that lead to whether a physician ultimately encourages or discourages NEHR participation is influenced by a variety of elements, including data privacy and security concerns, ease-of-use for patients, EHR system infrastructure, and potential administrative burden, just to name a few.^3,48,49^ With these findings, doctors can further enhance a positive influence on NEHR adoption by continuing to strengthen the doctor–patient relationship, have a comprehensive understanding of the country's NEHRs, address informed consent with patients who are enrolling in the NEHRs, and advocate for NEHRs to have an ethical, patient-centered approach while also critiquing the system if it does not sufficiently benefit the patients or the healthcare system.^3^

Participants are more trusting of their doctor's opinions, while being more wary of political stakeholder motives due to consistent beliefs that politicians do not always have the best interests of the public in their decision-making processes.^3^ These actors must exercise great care and responsibility when establishing and implementing a national EHR system, as trust within the system will be extremely difficult to both obtain and sustain without the conscientious effort of these actors.

### Policy recommendations

To increase public trust in NEHRs, it is essential to prioritize data security and privacy protections. Governments and healthcare institutions should implement strong encryption and anonymization techniques to safeguard patient data during storage and health data transmission.^43,50^ Clear data governance policies must be established to ensure that only authorized personnel have access to sensitive information.^43^

Individual autonomy and informed consent methods must also be held in high regard. Individuals should have the ability to choose whether they wish to participate in NEHRs through a well-defined opt-in or opt-out system.^51^ The consent process should be clear, accessible, and updated regularly to reflect any changes in policy or technology.^43^ Furthermore, patients should have control over their data, with the ability to manage permissions for different stakeholders, including healthcare providers, researchers, and government agencies.^3^

Transparency in healthcare system operations and regulatory oversight is another key factor in building public trust. Independent oversight bodies should be established to monitor NEHRs, ensuring compliance with data protection regulations and ethical standards.^52^ Regular transparency reports could be published, detailing system performance, potential data breaches, and the corrective actions taken.^20^ Additionally, transparency is crucial, as public communication efforts should regularly inform citizens about the security measures in place to protect their health data and any steps taken to mitigate potential risks.^3,20^

Public trust in NEHRs is also influenced by country-specific concerns, which must be addressed through tailored policy interventions. In Germany and the Netherlands, where past government actions have led to skepticism, stronger accountability measures and clearer communication strategies should be implemented. In Italy, public trust in national institutions is lower, but there is greater confidence in EU-led initiatives. Therefore, aligning national NEHR strategies with broader EU frameworks may help foster trust. In Switzerland, the current EHR system is fragmented and confounding for the average person, discouraging public participation. Simplifying the enrollment process and ensuring uniform communication across healthcare providers would help improve public trust and participation in the system.

Lastly, raising public awareness and improving digital literacy are essential for increasing acceptance of NEHRs. Public information campaigns could clearly explain the benefits, security provisions, and real-world impact of NEHRs.^3,20^ Educational programs could be developed to help individuals navigate and understand NEHR functionalities, ensuring that digital health tools are accessible to all residents and citizens of a country.^3^ Governments should also encourage citizen participation in policymaking by facilitating public consultations and feedback mechanisms. By involving the public in the decision-making process, policymakers can build more legitimate and trusted NEHRs.^20^

By implementing these policy recommendations, governments and healthcare stakeholders can strengthen public trust in NEHRs, leading to greater participation and improved healthcare outcomes. A comprehensive approach that includes security, transparency, tailored interventions, and public education will ensure the successful integration of NEHRs into national healthcare systems.

### Limitations

The limitations present in this study focus primarily on recruitment strategies and focus group sampling. One limitation was that, due to language barriers, half of the focus group interviews were not completed in the native language of the respective country. In France, Italy, and the Netherlands, only members of the public who could speak, at a minimum, conversational English were recruited for our study. This may have also contributed to a research bias, as, in the German-speaking countries, participants were more loquacious and proactive in suggesting new themes to the framework. Yet, in contrast, the non-German-speaking countries displayed less enthusiasm for introducing additional themes. Still, the inability to interview them in their native language was a limitation.

Another limitation was an issue with recruiting eligible participants in Italy, France, and the Netherlands who were citizens or residents of each country's population, respectively. While the methods of how this occurred are presently unknown to the research team, the recruitment flyers for the study were shared with individuals that were not citizens nor residents of the research countries. It is the belief of the research team that these individuals were attempting to take part in the study to receive the financial compensation offered to those who successfully took part in the focus group discussions. However, the research team was able to vet eligible participants with a high degree of accuracy and remove those who were not eligible to participate from the participant pool before focus group discussions began.

A third limitation of this study is the extent to which cultural transferability can be ascertained when comparing the results of the study countries to other countries. As the results showcase that each country produces its own unique results, therefore requiring additional research to determine another country's public trust in NEHRs, it is difficult to precisely ascertain how generalizable these results are to determining public trust in NEHRs in countries that were not included in the study. However, we argue that the methodology of our study is sufficiently generalizable for other researchers to utilize in their research on public trust in NEHRs in other countries and populations, if they so choose to.

A fourth limitation of this study concerns the underrepresentation of older adults in the focus groups. While adults 50 years old and older took part in the focus groups, the majority of participants were under the age of 50, with many of those participants being between the ages of 18 and 29. Future research should strive to access older adult communities to ascertain more viewpoints from their demographic on public trust in NEHRs.

An additional limitation was the lack of validation or pilot testing for the post-interview survey. However, as the objective of this survey was to primarily ascertain demographic data and previous experiences with EHRs, the research team believes that any potential issues from an absence of validation or pilot-testing are negligible for this type of data.

Lastly, a further limitation of the study was that in some countries, particularly the Netherlands, there were small sample sizes in certain focus groups. However, these smaller focus group discussions still bore more than adequate results, as these groups were fertile ground for in-depth discussions between participants, and between participants and moderators. Additionally, saturation was continuously reached by the fourth focus group in each country, which negated the need for further focus groups, even with larger sample sizes.

As the primary research team, KP and FG bring in over 12 years of trust research combined, potentially leading to preconceived notions on the subject of public trust. The expert academics and research partners enlisted in support of this research contributed to the mitigation of potential biases of the primary research team as they, being either native or long-term residents of the countries included in this study, provided crucial cultural and societal context to the study populations. These academic partners also provided a multitude of outside perspectives to the research and results, as these partners were academics in a variety of fields outside of the main research areas of the primary research team, and therefore assisted in assuaging potential preconceived outlooks from the primary research team.

## Conclusion

National EHR systems are an important asset to a country's healthcare system, and having public trust in these systems is imperative for their successful implementation and inducing significant public participation, allowing for the benefits of EHRs to be shared across society. Utilizing our frameworks, we can inform and guide policymakers and other healthcare stakeholders across multiple countries on how best to foster and maintain public trust in EHRs, through the context provided by the perspectives of their respective citizens and residents. This tailor-made approach will allow for more nuanced and personalized approaches to be taken to ensure that public trust, legitimacy, and participation will be in place for EHR implementation on a national level. This contribution allows for not only public trust in EHRs to be better understood in Austria, Germany, France, Italy, the Netherlands, and Switzerland, but also creates a foundation for further research into public trust in national EHR systems across other European countries, as well as those beyond the European continent.

## Supplemental Material

sj-docx-1-dhj-10.1177_20552076251333576 - Supplemental material for Understanding public trust in national electronic health record systems: A multi-national qualitative research study Supplemental material, sj-docx-1-dhj-10.1177_20552076251333576 for Understanding public trust in national electronic health record systems: A multi-national qualitative research study by Kimon Papadopoulos, Elske Ammenwerth, Guillaume Lame, Nina Stahl, Verena Struckmann, Viktor von Wyl and Felix Gille in DIGITAL HEALTH

sj-docx-2-dhj-10.1177_20552076251333576 - Supplemental material for Understanding public trust in national electronic health record systems: A multi-national qualitative research study Supplemental material, sj-docx-2-dhj-10.1177_20552076251333576 for Understanding public trust in national electronic health record systems: A multi-national qualitative research study by Kimon Papadopoulos, Elske Ammenwerth, Guillaume Lame, Nina Stahl, Verena Struckmann, Viktor von Wyl and Felix Gille in DIGITAL HEALTH

sj-docx-3-dhj-10.1177_20552076251333576 - Supplemental material for Understanding public trust in national electronic health record systems: A multi-national qualitative research study Supplemental material, sj-docx-3-dhj-10.1177_20552076251333576 for Understanding public trust in national electronic health record systems: A multi-national qualitative research study by Kimon Papadopoulos, Elske Ammenwerth, Guillaume Lame, Nina Stahl, Verena Struckmann, Viktor von Wyl and Felix Gille in DIGITAL HEALTH

sj-pdf-4-dhj-10.1177_20552076251333576 - Supplemental material for Understanding public trust in national electronic health record systems: A multi-national qualitative research study Supplemental material, sj-pdf-4-dhj-10.1177_20552076251333576 for Understanding public trust in national electronic health record systems: A multi-national qualitative research study by Kimon Papadopoulos, Elske Ammenwerth, Guillaume Lame, Nina Stahl, Verena Struckmann, Viktor von Wyl and Felix Gille in DIGITAL HEALTH

sj-xlsx-5-dhj-10.1177_20552076251333576 - Supplemental material for Understanding public trust in national electronic health record systems: A multi-national qualitative research study Supplemental material, sj-xlsx-5-dhj-10.1177_20552076251333576 for Understanding public trust in national electronic health record systems: A multi-national qualitative research study by Kimon Papadopoulos, Elske Ammenwerth, Guillaume Lame, Nina Stahl, Verena Struckmann, Viktor von Wyl and Felix Gille in DIGITAL HEALTH
