# Supplementary figures and images for: Understanding public trust in national electronic health record systems: A multi-national qualitative research study
Source: Digit Health. 2025 Apr 3;11:20552076251333576. doi: 10.1177/20552076251333576 (PMC11970066; doi:10.1177/20552076251333576)

**Appendix A:** Example of Flinga Online Whiteboard from an Italian focus group


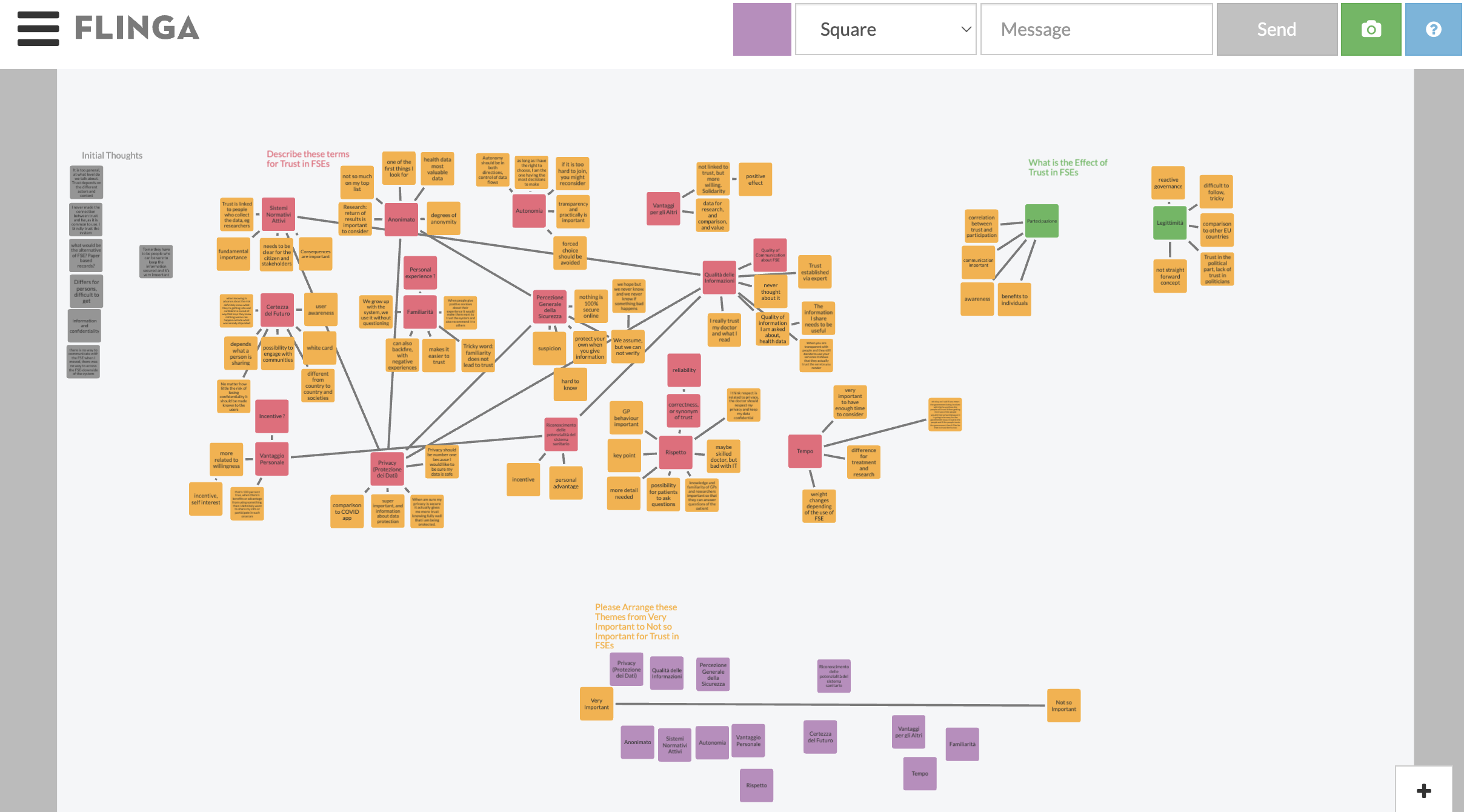

Supplement: sj-docx-1-dhj-10.1177_20552076251333576 - Supplemental material for Understanding public trust in national electronic health record systems: A multi-national qualitative research study [file sj-docx-1-dhj-10.1177_20552076251333576.docx]
