# Supplementary material for: Understanding public trust in national electronic health record systems: A multi-national qualitative research study
Source: Digit Health. 2025 Apr 3;11:20552076251333576. doi: 10.1177/20552076251333576 (PMC11970066; doi:10.1177/20552076251333576)
Supplement: sj-docx-2-dhj-10.1177_20552076251333576 - Supplemental material for Understanding public trust in national electronic health record systems: A multi-national qualitative research study [file sj-docx-2-dhj-10.1177_20552076251333576.docx]

**Appendix B:** Participant Demographics

Austria

| Baseline characteristic | Participants  (n = 17) | |
| --- | --- | --- |
|  | *n* | % |
| Gender  Female | 12 | 70.6 |
| Male | 5 | 29.4 |
| No Response | 0 | 0 |
| Age |  |  |
| 18 - 29 | 6 | 35.3 |
| 30 - 49 | 8 | 47.1 |
| 50 - 64 | 2 | 11.8 |
| 65+ | 0 | 0 |
| No Response | 1 | 5.8 |
| Country of Origin |  |  |
| Austria | 13 | 76.6 |
| Germany | 2 | 11.8 |
| USA | 1 | 5.8 |
| No Response | 1 | 5.8 |
| Current Region of Residence |  |  |
| Brunn am Gebirge | 1 | 8.3 |
| Absam | 2 | 11.8 |
| Wien | 3 | 17.5 |
| Hall in Tirol | 2 | 11.8 |
| Innsbruck | 2 | 11.8 |
| Zisterndorf | 1 | 8.3 |
| Wattens | 1 | 8.3 |
| Salzburg | 1 | 8.3 |
| Thaur | 1 | 8.3 |
| Ried im Innkreis | 1 | 8.3 |
| Bezirk | 1 | 8.3 |
| No Response | 0 | 0 |
| Highest Educational Level |  |  |
| Berufsbildende mittlere oder höhere Schule | 2 | 11.8 |
| Allgemeinbildende höhere Schule | 3 | 17.5 |
| Hochschulabschluss | 10 | 58.9 |
| Doktorat | 2 | 11.8 |
| No Response  Employment Status | 0 | 0 |
| Unemployed/Retired | 0 | 0 |
| Student | 4 | 23.4 |
| Employed | 13 | 76.6 |

Germany

| Baseline characteristic | Participants  (n = 12) | |
| --- | --- | --- |
|  | *n* | % |
| Gender  Female | 7 | 58.3 |
| Male | 5 | 41.7 |
| No Response | 0 | 0 |
| Age |  |  |
| 18 - 29 | 5 | 41.7 |
| 30 - 49 | 3 | 25.0 |
| 50 - 64 | 0 | 0 |
| 65+ | 1 | 8.3 |
| No Response | 3 | 25.0 |
| Country of Origin |  |  |
| Germany | 10 | 83.4 |
| Lithuania | 1 | 8.3 |
| No Response | 1 | 8.3 |
| Current Region of Residence |  |  |
| Berlin | 5 | 41.7 |
| Baden-Wurttemburg | 1 | 8.3 |
| Brandenburg | 1 | 8.3 |
| Nordrhein-Westfalen | 4 | 33.3 |
| No Response | 0 | 0 |
| Highest Educational Level |  |  |
| Obligatory School | 1 | 8.3 |
| Berufslehre | 1 | 8.3 |
| Hochschulabschluss | 10 | 83.4 |
| No Response  Employment Status | 0 | 0 |
| Unemployed/Retired | 0 | 0 |
| Student | 3 | 25.0 |
| Employed | 8 | 66.7 |
| No Response | 1 | 8.3 |

France

| Baseline characteristic | Participants  (n =12) | |
| --- | --- | --- |
|  | *n* | % |
| Gender  Female | 6 | 50 |
| Male | 6 | 50 |
| No Response | 0 | 0 |
| Age |  |  |
| 18 - 29 | 8 | 66.7 |
| 30 - 49 | 4 | 33.3 |
| 50 - 64 | 0 | 0 |
| 65+ | 0 | 0 |
| No Response | 0 | 0 |
| Country of Origin |  |  |
| France | 8 | 66.7 |
| Mauritius | 1 | 8.3 |
| Switzerland | 1 | 8.3 |
| Mexico | 1 | 8.3 |
| Spain | 1 | 8.3 |
| No Response | 0 | 0 |
| Current Region of Residence |  |  |
| Ile-de-France | 4 | 33.3 |
| Auvergne-Rhône-Alpes | 3 | 25 |
| Bretagne | 1 | 8.3 |
| Outside of France | 4 | 33.3 |
| No Response | 0 | 0 |
| Highest Educational Level |  |  |
| L’université / Les Grandes écoles | 10 | 83.3 |
| Le baccalauréat | 2 | 16.7 |
| No Response  Employment Status | 0 | 0 |
| Unemployed/Retired | 0 | 0 |
| Student | 3 | 25 |
| Employed | 9 | 75 |
| No Response | 0 | 0 |

Italy

| Baseline characteristic | Participants  (n = 26) | |
| --- | --- | --- |
|  | *n* | % |
| Gender  Female | 10 | 38 |
| Male | 15 | 58 |
| No Response | 1 | 4 |
| Age |  |  |
| 18 - 29 | 15 | 58 |
| 30 - 49 | 9 | 34 |
| 50 - 64 | 0 | 0 |
| 65+ | 0 | 0 |
| No Response | 2 | 8 |
| Country of Origin |  |  |
| Italy | 18 | 69 |
| United Kingdom | 3 | 11 |
| Switzerland | 1 | 4 |
| USA | 2 | 8 |
| France | 1 | 4 |
| No Response | 1 | 4 |
| Current Region of Residence |  |  |
| Piemonte | 3 | 11 |
| Lazio | 5 | 19 |
| Romagna | 1 | 4 |
| Lombardia | 1 | 4 |
| Abruzzo | 1 | 4 |
| Calabria | 2 | 8 |
| Toscana | 1 | 4 |
| Sicilia | 1 | 4 |
| Campania | 1 | 4 |
| Basilicata | 1 | 4 |
| Outside of Italy | 8 | 30 |
| No Response | 1 | 4 |
| Highest Educational Level |  |  |
| Dottorato di ricerca | 6 | 23 |
| Laurea Magistrale | 7 | 27 |
| Laurea Triennale | 12 | 46 |
| No Response  Employment Status | 1 | 4 |
| Unemployed/Retired | 0 | 0 |
| Student | 7 | 27 |
| Employed | 18 | 69 |
| No Response | 1 | 4 |

The Netherlands

| Baseline characteristic | Participants  (n = 9) | |
| --- | --- | --- |
|  | *n* | % |
| Gender  Female | 6 | 66.7 |
| Male | 2 | 22.2 |
| No Response | 1 | 11.1 |
| Age |  |  |
| 18 - 29 | 6 | 66.7 |
| 30 - 49 | 2 | 22.2 |
| 50 - 64 | 0 | 0 |
| 65+ | 0 | 0 |
| No Response | 1 | 11.1 |
| Country of Origin |  |  |
| The Netherlands | 3 | 33.3 |
| Russia | 1 | 11.1 |
| Belgium | 2 | 22.2 |
| Italy | 1 | 11.1 |
| Slovakia | 1 | 11.1 |
| No Response | 1 | 11.1 |
| Current Region of Residence |  |  |
| Noord-Holland | 4 | 44.4 |
| Limburg | 1 | 11.1 |
| Outside of the Netherlands | 3 | 33.3 |
| No Response | 1 | 11.1 |
| Highest Educational Level |  |  |
| Higher Education | 8 | 88.9 |
| No Response  Employment Status | 1 | 11.1 |
| Unemployed/Retired | 0 | 0 |
| Student | 2 | 22.2 |
| Employed | 6 | 66.7 |
| No Response | 1 | 11.1 |

Switzerland

| Baseline characteristic | Participants  (n = 13) | |
| --- | --- | --- |
|  | *n* | % |
| Gender  Female | 7 | 53.8 |
| Male | 6 | 46.2 |
| No Response | 0 | 0 |
| Age |  |  |
| 18 - 29 | 5 | 38.5 |
| 30 - 49 | 2 | 15.4 |
| 50 - 64 | 2 | 15.4 |
| 65+ | 1 | 7.7 |
| No Response | 3 | 23.1 |
| Country of Origin |  |  |
| Switzerland | 3 | 23.1 |
| Germany | 3 | 23.1 |
| Russia | 1 | 7.7 |
| No Response | 6 | 46.2 |
| Current Region of Residence |  |  |
| Zurich | 6 | 46.2 |
| Aargau | 3 | 23.1 |
| Luzern | 1 | 7.7 |
| Bern | 2 | 15.4 |
| Nidwalden | 1 | 7.7 |
| No Response | 0 | 0 |
| Highest Educational Level |  |  |
| Doktorat | 2 | 15.4 |
| Hochschulabschluss | 4 | 30.1 |
| Maturität | 4 | 30.1 |
| Höhere Fachschulen | 1 | 7.7 |
| Lehre | 1 | 7.7 |
| No Response  Employment Status | 1 | 7.7 |
| Unemployed/Retired | 2 | 15.4 |
| Student | 4 | 30.1 |
| Employed | 6 | 46.2 |
| No Response | 1 | 7.7 |
