# Supplementary material for: Understanding public trust in national electronic health record systems: A multi-national qualitative research study
Source: Digit Health. 2025 Apr 3;11:20552076251333576. doi: 10.1177/20552076251333576 (PMC11970066; doi:10.1177/20552076251333576)
Supplement: sj-docx-3-dhj-10.1177_20552076251333576 - Supplemental material for Understanding public trust in national electronic health record systems: A multi-national qualitative research study [file sj-docx-3-dhj-10.1177_20552076251333576.docx]

**Appendix C:** Country-adapted Frameworks

Native Language Frameworks

Austrian Framework - German

| **Causal Theme** | **Contextual Definition** |
| --- | --- |
| Eigenmotivierte Einhaltung von Gesetzen | Wenn diejenigen die ELGA benutzen sich eigenmotiviert an Gesetze und Regeln halten, dann vertraue ich der ELGA. |
| Datenanonymität in der Forschung | Wenn meine Daten nur anonymisiert für Forschung weitergegeben werden, dann vertraue ich der ELGA. |
| Entscheidungskontrolle über die ELGA | Wenn ich eigenständig und frei entscheiden kann, ob ich die ELGA benutzen möchten, dann vertraue ich der ELGA. |
| Positivie Erfahrung mit der ELGA | Wenn ich positive Erfahrungen mit der ELGA hatten, dann vertraue ich ELGA. |
| Datenschutz | Wenn die ELGA meine Daten schützt, dann vertraue ich der ELGA. |
| Generelles Gefühl der Sicherheit bezüglich der ELGA | Wenn ich die ELGA als sicher erachten, dann vertraue ich der ELGA. |
| Ein gesellschaftlicher Nutzen entsteht duch die ELGA | Wenn durch den Gebrauch von ELGA ein Nutzen für die Gesellschaft entsteht, dann vertraue ich der ELGA. |
| Gewsissheit über die zukünftige Risiken bezüglich der ELGA | Wenn diejenigen die ELGA benutzen ihr Bestes geben, um mögliche Risiken im Gebrauch von ELGA vorherzusehen, dann vertraue ich ELGA. |
| Ein perönlicher Nutzen entsteht duch die ELGA | Wenn ich einen persönlichen Nutzen durch die ELGA habe, dann vertrauen ich der ELGA. |
| Erkennbares Leistungspotential der ELGA | Wenn ich ein Leistungspotential in der ELGA erkenne, dass zu erreichen, wofür Sie eingeführt wurde, dann vertraue ich der ELGA. |
| Respektvoller Umgang zwischen Patient und Gesundheitsversorger | Wenn ich und mein Gesundheitsversorger uns gegenseitig respektieren, dann vertraue ich der ELGA. |
| Ein System Nutzen entsteht duch die ELGA | Wenn durch den Gebrauch von ELGA ein Nutzen für das Gesundheitssystem entsteht, dann vertrauen ich ELGA. |
| Transparente Kommunikation über die ELGA | Wenn über ELGA transparent kommuniziert wird, dann vertraue ich ELGA |
| Verlässlichkeit der ELGA | Wenn die ELGA ein verlässliches System ist, dann vertraue ich der ELGA. |
| Bedenkzeit für mein Einverstäntnis für die ELGA | Wenn ich aussreichend Bedenkzeit für mein Einverstäntnis habe, dann vertraue ich ELGA. |
| Zeitersparniss duch die ELGA | Wenn ELGA zu Zeitersparnissen im Gesundheitssystem führt, dann vertrauen sie ELGA. |
| **Effect Theme** | **Contextual Definition** |
| Die ELGA wird benutzt | Wenn ich der ELGA vertraue, dann nutze ich ELGA. |
| Die ELGA ist zulässig | Wenn ich der ELGA vertraue, dann ist die ELGA zulässig. |

German Framework - German

| **Causal Theme** | **Contextual Definition** |
| --- | --- |
| Eigenmotivierte Einhaltung von Gesetzen | Wenn diejenigen die ePA benutzen sich eigenmotiviert an Gesetze und Regeln halten, dann vertraue ich der ePA. |
| Datenschutz | Wenn die ePA meine Daten schützt, dann vertraue ich der ePA. |
| Ein perönlicher Nutzen entsteht duch die ePA | Wenn ich einen persönlichen Nutzen durch die ePA habe, dann vertrauen ich der ePA. |
| Ein gesellschaftlicher Nutzen entsteht duch die ePA | Wenn durch den Gebrauch von ePA ein Nutzen für die Gesellschaft entsteht, dann vertraue ich der ePA. |
| Sichergestellte Einführung der ePA | Wenn die ePA Einführung sichergestellt ist, verteaue ich der ePA. |
| Vertrauen in die ePA des medizinischen Personal | Wenn medizinisches Personal (z.B. Hausärztin) der ePA vertraut, dann vertraue ich der ePA. |
| Datenanonymität in der Forschung | Wenn meine Daten nur anonymisiert für Forschung weitergegeben werden, dann vertraue ich der ePA. |
| Bedenkzeit für mein Einverstäntnis für die ePA | Wenn ich aussreichend Bedenkzeit für mein Einverstäntnis habe, dann vertraue ich ePA. |
| Nutzerfreundlichkeit der ePA | Wenn ePA nutzerfreundlich ist, dann vertraue ich der ePA. |
| Erkennbares Leistungspotential der ePA | Wenn ich ein Potential in der ePA erkenne, dass zu erreichen, wofür Sie eingeführt wurde, dann vertraue ich der ePA. |
| Entscheidungskontrolle über die ePA | Wenn ich eigenständig und frei entscheiden kann, ob ich die ePA benutzen möchten, dann vertraue ich der ePA. |
| Datenqualität in der ePA | Wenn die Daten in der ePA korrekt sind, dann vertraue ich der ePA. |
| Sorgfältiger Datenumgang innerhalb der ePA | Wenn mit ihren Daten sorgfältig umgegangen wird, dann vertraue ich der ePA. |
| Übertragbarkeit der ePA zwischen Versorgern | Wenn die ePA übertragbar ist zwischen Versorgern, dann vertraue ich der ePA. |
| Einfach verständliche ePA. | Wenn die ePA einfach verständlich ist, dann vertraue ich der ePA. |
| Generelles Gefühl der Sicherheit bezüglich der ePA | Wenn ich die ePA als sicher erachten, dann vertraue ich der ePA. |
| **Effect Theme** | **Contextual Definition** |
| Die ePA wird benutzt | Wenn ich der ePA vertraue, dann nutze ich die ePA. |
| Die ePA wird akzeptiert | Wenn ich der ePA vertraue, dann akzeptiere ich die ePA. |
| ePA Markenbildung durch Nutzung | Wenn ich der ePA vertraue, kommt es durch die breite Nutzung zu einer Markenbildung. |
| Forschungsnutzen der ePA | Wenn ich der ePA vertraue, dann kann ePA für Forschung benutzt werden. |

French Framework - French

| **Causal Theme** | **Contextual Definition** |
| --- | --- |
| Système Réglementaires Actifs | Si les acteurs du système de santé qui utilisent les DMP se conforment activement aux lois et aux règles, vous avez alors davantage confiance dans les DMP. |
| L'anonymat dans la recherche | Si les médecins anonymisent vos données pour la recherche, vous leur faites davantage confiance. |
| Liberté de participation | Si vous êtes libre d'utiliser les DMP, vous leur faites davantage confiance. |
| Familiarité | Si vous avez eu une expérience positive avec les DMP, vous leur faites confiance. |
| Perception générale de la sécurité | Si vous considérez que les DMP sont sûrs, vous leur faites confiance. |
| Transparence | Lorsque des informations fiables sont communiquées de manière transparente sur les DMP, la confiance dans les DMP est plus grande. |
| Protection des données | Lorsque les DMP protègent votre vie privée, vous leur faites confiance. |
| Une relation médecin-patient respectueuse | Si vous entretenez une relation médecin-patient respectueuse, vous faites davantage confiance aux DMP. |
| Temps de prise de décision | Si l'on vous laisse suffisamment de temps pour décider si vous voulez participer ou non aux DMP, vous faites davantage confiance aux DMP. |
| **Effect Theme** | **Contextual Definition** |
| La participation | Si vous faites confiance aux DMP, vous utilisez les DMP. |
| Pouvoir d'agir | Si vous faites confiance aux DMP, vous permettez aux décideurs politiques de les mettre en œuvre en toute légitimité. |

Italian Framework - Italian

| **Causal Theme** | **Contextual Definition** |
| --- | --- |
| Sistemi Normativi Attivi | Se gli operatori del sistema sanitario usano il FSE seguendo le leggi e i protocolli in vigore, allora si ha fiducia nel FSE. |
| Anonimato nella Ricerca | Se il FSE anonimizza i dati durante la ricerca, allora si ha fiducia nel FSE. |
| Autonomia | Se si puo' decidere se usare il FSE o meno, allora si ha fiducia nel FSE. |
| Consapevolezza dei rischi | Se coloro che utilizzano il FSE fanno del loro meglio per prevedere i possibili rischi nell'uso dei FSE, allora si ha fiducia nel FSE. |
| Esperienza positiva | Se si é avuta un'esperienza positiva con l’FSE, allora si ha fiducia nel FSE |
| Percezione Generale della Sicurezza | Se si considera il FSE sicuro, allora si ha fiducia nel FSE |
| Trasparenza | Se vengono comunicate informazioni oneste e veritiere sul FSE, allora si ha fiducia nel FSE |
| Protezione dei Dati | Se il FSE protegge la privacy, allora si ha fiducia nel FSE |
| Rapporto operatore sanitario-paziente | Se vi è rispetto reciproco tra paziente e operatore sanitario, allora si ha fiducia nel FSE |
| Tempo per prendere decisioni | Se viene dato tempo sufficiente per decidere se si voglia o meno participare al FSE, allora si ha fiducia nel FSE |
| **Effect Theme** | **Contextual Definition** |
| Partecipazione | Se ci si fida del FSE, allora lo si usa |
| Attribuzione di facoltà | Se ci si fida del FSE, si dà alle autorità il potere di implementare il FSE in modo legitimo |

Dutch Framework - Dutch

| **Causal Theme** | **Contextual Definition** |
| --- | --- |
| Actieve Regelgevingssystemen | Als actoren in de gezondheidszorg, die gebruik maken van EPDs, zich actief houden aan wetten en regelgeving, dan heeft u meer vertrouwen in EPDs. |
| Anonimiteit in Onderzoek | Als EPDs uw gegevens anonimiseren tijdens het onderzoek, dan vertrouwt u EPDs meer. |
| Vrijheid om Deel te Nemen | Als u de vrijheid heeft om zelf te beslissen of u mee wilt doen aan EPDs, dan vertrouw u EPDs meer. |
| Maatschappelijk voordeel | Als het gebruik van EPDs anderen en het systeem ten goede komt, vertrouwt u EPDs meer. |
| Risicobewustzijn | Als diegenen die EPDs gebruiken op de hoogte zijn van mogelijke risico's van het gebruik van EPDs, dan vertrouw u EPDs meer. |
| Positieve Ervaring | Als u een positieve ervaring had met EPDs, dan vertrouwt u EPDs meer. |
| Gegevensbescherming | Als u EPDs als veilig en betrouwbaar beschouwt, dan vertrouwt u EPDs meer. |
| Transparante Communicatie | Als er eerlijke en waarheidsgetrouwe informatie over EPDs wordt gecommuniceerd, dan is er ook meer vertrouwen in EPDs. |
| Privacy | Wanneer EPDs uw privacy beschermen, vertrouwt u EPDs meer. |
| Bekwaamheid gezondheidszorgsysteem | Als u in EPDs een potentieel ziet om te bereiken waarvoor het is ingevoerd, dan vertrouwt u EPDs. |
| Respect in de Relatie Arts - Patiënt | Wanneer u en uw zorgverlener elkaar respecteren, vertrouwt u EDPs meer. |
| Tijd voor besluitvorming | Als u voldoende tijd krijgt om te beslissen of u aan EPDs wilt deelnemen, vertrouwt u EPDs meer. |
| **Effect Theme** | **Contextual Definition** |
| Deelname | Als u EPD's vertrouwt, dan gebruikt u EPDs. |
| Handelings-bevoegdheid | Als u EPDs vertrouwt, geeft u autoriteitsfiguren de macht om EPDs legitiem te implementeren. |

Swiss Framework - German

| **Causal Theme** | **Contextual Definition** |
| --- | --- |
| Datenanonymität in der Forschung | Wenn meine Daten nur anonymisiert für Forschung weitergegeben werden, dann vertraue ich dem EPD. |
| Datenschutz | Wenn das EPD meine Daten schützt, dann vertraue ich dem EPD. |
| Eigenmotivierte Einhaltung von Gesetzen | Wenn diejenigen das EPD benutzen sich eigenmotiviert an Gesetze und Regeln halten, dann vertraue ich dem EPD. |
| Einheitliche Kommunikation über das EPD | Wenn über das EPD einheitlich kommuniziert wird, dann vertrauen ich dem EPD. |
| Empfehlung Anderer das EPD zu nutzen | Wenn Andere mir das EPD empfehlen, dann vertraue ich dem EPD. |
| Eigene Entscheidungs-kompetenz über das EPD | Wenn ich eigenständig und frei entscheiden kann, ob ich das EPD benutzen möchte, dann vertraue ich dem EPD. |
| Generelles Gefühl der Sicherheit bezüglich dem EPD | Wenn ich das EPD als sicher erachten, dann vertraue ich dem EPD. |
| Kompetenz des Gesundheitssystems im Umgang mit dem EPD | Wenn Sie das Gesundheitssystem als kompetent im Umgang mit dem EPD einschätzen, dann vertrauen sie dem EPD |
| Ein gesellschaftlicher Nutzen entsteht duch das EPD | Wenn durch den Gebrauch von dem EPD ein Nutzen für die Gesellschaft entsteht, dann vertraue ich dem EPD. |
| Ein perönlicher Nutzen entsteht duch das EPD | Wenn ich einen persönlichen Nutzen durch das EPD habe, dann vertrauen ich dem EPD. |
| Respektvoller Umgang zwischen Patient und Gesundheitsversorger | Wenn Gesundheitsversorger respektvoll mit mir und meinen Daten umgehen, dann vertraue ich dem EPD |
| Sensibilisierung der Bevölkerung über den konkreten Nutzen des EPDs. | Wenn der Bevölkerung der konkrete Nutzen der EPD erklärt wird, dann vertraue ich der EPD. |
| Zeitnahe Einführung der EPD | Wenn das EPD zeitnah eingeführt wird, dann vertraue ich dem EPD. |
| **Effect Theme** | **Contextual Definition** |
| Das EPD wird benutzt | Wenn ich dem EPD vertraue, dann nutze ich das EPD. |
| Das EPD wird akzeptiert | Wenn ich dem EPD vertraue, dann akzeptiere ich das EPD. |
| Teilhabe am EPD | Wenn ich dem EPD vertrauen, dann haben ich ein Gefühl der Teilhabe. |
| Empfehlung des EPD an Andere | Wenn Sie dem EPD vertrauen, dann empfehlen ich das EPD an andere. |

English Translated Frameworks

Austrian Framework - English

| **Causal Theme** | **Contextual Definition** |
| --- | --- |
| Self-motivated compliance with laws | If those who use ELGA are self-motivated to adhere to laws and rules, then I trust ELGA. |
| Data Anonymity in Research | If my data is only passed on anonymously for research purposes, then I trust ELGA. |
| decision-making control over the ELGA | If I can decide independently and freely whether I want to use ELGA, then I trust ELGA. |
| Positive experience with ELGA | If I have had positive experiences with ELGA, then I trust ELGA. |
| data protection | If ELGA protects my data, then I trust ELGA. |
| General feeling of security regarding ELGA | If I consider ELGA to be safe, then I trust ELGA. |
| A social benefit is created by ELGA | If the use of ELGA creates a benefit for society, then I trust ELGA. |
| Certainty about future risks regarding ELGA | If those who use ELGA do their best to anticipate possible risks in using ELGA, then I trust ELGA. |
| A personal benefit arises from the ELGA | If I personally benefit from ELGA, then I trust ELGA. |
| Recognizable performance potential of ELGA | If I see potential in ELGA to achieve what it was introduced for, then I trust ELGA. |
| Respectful interaction between patient and healthcare provider | If I and my healthcare provider respect each other, then I trust ELGA. |
| A system benefit is created by the ELGA | If the use of ELGA creates a benefit for the healthcare system, then I trust ELGA. |
| Transparent communication via ELGA | If communication about ELGA is transparent, then I trust ELGA |
| reliability of ELGA | If ELGA is a reliable system, then I trust ELGA. |
| Consideration period for my consent for the ELGA | If I have enough time to consider my consent, then I trust ELGA. |
| Time savings through ELGA | If ELGA saves time in the healthcare system, then trust ELGA. |
| **Effect Theme** | **Contextual Definition** |
| The ELGA is used | If I trust ELGA, then I use ELGA. |
| The ELGA is permissible | If I trust the ELGA, then the ELGA is permissible. |

German Framework - English

| **Causal Theme** | **Contextual Definition** |
| --- | --- |
| Self-motivated compliance with laws | If those who use the ePA are self-motivated to adhere to laws and rules, then I trust the ePA. |
| data protection | If the ePA protects my data, then I trust the ePA. |
| A personal benefit is created by the ePA | If I personally benefit from the ePA, then I trust the ePA. |
| A social benefit is created by the ePA | If the use of ePA creates a benefit for society, then I trust the ePA. |
| Ensured introduction of the ePA | If the ePA introduction is ensured, I will support the ePA. |
| Trust in the ePA of medical staff | If medical staff (e.g. family doctor) trusts the ePA, then I trust the ePA. |
| Data Anonymity in Research | If my data is only passed on anonymously for research purposes, then I trust the ePA. |
| Consideration period for my consent for the ePA | If I have enough time to consider my consent, then I trust ePA. |
| user-friendliness of the ePA | If ePA is user-friendly, then I trust the ePA. |
| Recognizable performance potential of the ePA | If I see potential in the ePA to achieve what it was introduced for, then I trust the ePA. |
| decision control via the ePA | If I can decide independently and freely whether I want to use the ePA, then I trust the ePA. |
| Data quality in the ePA | If the data in the ePA is correct, then I trust the ePA. |
| Careful data handling within the ePA | If your data is handled carefully, then I trust the ePA. |
| Transferability of the ePA between providers | If the ePA is transferable between providers, then I trust the ePA. |
| Easy-to-understand ePA. | If the ePA is easy to understand, then I trust the ePA. |
| General feeling of security regarding the ePA | If I consider the ePA to be secure, then I trust the ePA. |
| **Effect Theme** | **Contextual Definition** |
| The ePA is used | If I trust the ePA, then I use the ePA. |
| The ePA is accepted | If I trust the ePA, then I accept the ePA. |
| ePA branding through use | If I trust the ePA, its widespread use will lead to brand building. |
| research benefits of the ePA | If I trust the ePA, then the ePA can be used for research. |

French Framework – English

| **Causal Theme** | **Contextual Definition** |
| --- | --- |
| Active Regulatory Systems | If the health system actors who use DMPs actively comply with laws and rules, then you have more confidence in the DMPs. |
| Anonymity in research | If doctors anonymize your data for research, you trust them more. |
| Freedom of participation | If you are free to use DMPs, you trust them more. |
| Familiarity | If you have had a positive experience with DMPs, you trust them. |
| General perception of safety | If you consider DMPs to be safe, you trust them. |
| Transparency | When reliable information is communicated transparently about DMPs, trust in DMPs is greater. |
| Data protection | When DMPs protect your privacy, you trust them. |
| A respectful doctor-patient relationship | If you have a respectful doctor-patient relationship, you trust DMPs more. |
| Decision making time | If you are given enough time to decide whether or not to participate in DMPs, you trust DMPs more. |
| **Effect Theme** | **Contextual Definition** |
| Participation | If you trust DMPs, you use DMPs. |
| Power to act | If you trust DMPs, you enable policy makers to implement them legitimately. |

Italian Framework – English

| **Causal Theme** | **Contextual Definition** |
| --- | --- |
| Active Regulatory Systems | If healthcare workers use the FSE following the laws and protocols in force, then the FSE is trusted. |
| Anonymity in Research | If the FSE anonymizes data during research, then you have trust in the FSE. |
| Autonomy | If you can decide whether to use the ESF or not, then you have confidence in the ESF. |
| Risk awareness | If those who use the FSE do their best to anticipate the possible risks in using the FSE, then the FSE is trusted. |
| Positive experience | If you have had a positive experience with the FSE, then you have confidence in the FSE. |
| General Perception of Safety | If you consider the ESF safe, then you have confidence in the ESF. |
| Transparency | If honest and truthful information is provided about the FSE, then there is trust in the FSE. |
| Data Protection | If the FSE protects privacy, then you trust the FSE |
| Healthcare provider-patient relationship | If there is mutual respect between patient and healthcare professional, then there is trust in the FSE |
| Time to make decisions | If you are given enough time to decide whether or not you want to participate in the ESF, then you have confidence in the ESF. |
| **Effects Theme** | **Contextual Definition** |
| Participation | If you trust the ESF, then you use it |
| Attribution of powers | If you trust the ESF, you give the authorities the power to implement the ESF legitimately. |

Dutch Framework – English

| **Causal Theme** | **Contextual Definition** |
| --- | --- |
| Active Regulatory Systems | If healthcare actors who use EHRs actively adhere to laws and regulations, you will have more confidence in EHRs. |
| Anonymity in Research | If EHRs anonymize your data during research, you will trust EHRs more. |
| Freedom to Participate | If you have the freedom to decide for yourself whether you want to participate in EHRs, you will trust EHRs more. |
| Social benefit | If using EHRs benefits others and the system, you will trust EHRs more. |
| Risk awareness | If those who use EHRs are aware of the potential risks of using EHRs, you will trust EHRs more. |
| Positive Experience | If you had a positive experience with EHRs, you will trust EHRs more. |
| Data protection | If you perceive EHRs as safe and reliable, you will trust EHRs more. |
| Transparent Communication | If honest and truthful information is communicated about EHRs, there will also be more trust in EHRs. |
| Privacy | When EHRs protect your privacy, you trust EHRs more. |
| Healthcare system competence | If you see the potential in EHRs to achieve what they were introduced for, then you trust EHRs. |
| Respect in the Doctor-Patient Relationship | When you and your healthcare provider respect each other, you will trust EDPs more. |
| Time for decision making | If you are given enough time to decide whether to participate in EHRs, you will trust EHRs more. |
| **Effect Theme** | **Contextual Definition** |
| Participation | If you trust EHRs, then use EHRs. |
| Authority to act | When you trust EHRs, you empower authority figures to implement EHRs legitimately. |

Swiss Framework – English

| **Causal Theme** | **Contextual Definition** |
| --- | --- |
| Data Anonymity in Research | If my data is only passed on anonymously for research purposes, then I trust the EPD. |
| data protection | If the EPD protects my data, then I trust the EPD. |
| Self-motivated compliance with laws | If those who use the EPD are self-motivated to adhere to laws and rules, then I trust the EPD. |
| Uniform communication about the EPD | If the EPD is communicated consistently, then I trust the EPD. |
| Recommend others to use the EPD | If others recommend the EPD to me, then I trust the EPD. |
| Own decision-making authority regarding the EPD | If I can decide independently and freely whether I want to use the EPD, then I trust the EPD. |
| General feeling of security regarding the EPD | If I consider the EPD to be safe, then I trust the EPD. |
| Competence of the health system in dealing with the EPD | If you consider the health system to be competent in dealing with the EPD, then you trust the EPD |
| A social benefit is created by the EPD | If the use of the EPD creates a benefit for society, then I trust the EPD. |
| A personal benefit arises from the EPD | If I personally benefit from the EPD, then I trust the EPD. |
| Respectful interaction between patient and healthcare provider | If healthcare providers treat me and my data with respect, then I trust the EPD |
| Raising public awareness about the concrete benefits of the EPD. | If the concrete benefits of the EPD are explained to the population, then I trust the EPD. |
| Timely introduction of the EPD | If the EPD is introduced in a timely manner, then I trust the EPD. |
| **Effect Theme** | **Contextual Definition** |
| The EPD is used | If I trust the EPD, then I use the EPD. |
| The EPD is accepted | If I trust the EPD, then I accept the EPD. |
| participation in the EPD | If I trust the EPD, then I have a feeling of participation. |
| Recommendation of the EPD to Others | If you trust the EPD, then I will recommend the EPD to others. |
